# Systemic Importance and Risk Characteristics of Banks Based on a Multi-Layer Financial Network Analysis

**DOI:** 10.3390/e26050378

**Published:** 2024-04-29

**Authors:** Qianqian Gao, Hong Fan, Chengyang Yu

**Affiliations:** 1 School of Financial Technology, Shanghai Lixin University of Accounting and Finance, Shanghai 201209, China; gaoqianqian@lixin.edu.cn; 2 Glorious Sun School of Business and Management, Donghua University, Shanghai 200051, China; 2221354@mail.dhu.edu.cn

**Keywords:** systemic risk, PageRank algorithm, network, centrality, risk exposure

## Abstract

Domestic and international risk shocks have greatly increased the demand for systemic risk management in China. This paper estimates China’s multi-layer financial network based on multiple financial relationships among banks, assets, and firms, using China’s banking system data in 2021. An improved PageRank algorithm is proposed to identify systemically important banks and other economic sectors, and a stress test is conducted. This study finds that China’s multi-layer financial network is sparse, and the distribution of transactions across financial markets is uneven. Regulatory authorities should support economic recovery and adjust the money supply, while banks should differentiate competition and manage risks better. Based on the PageRank index, this paper assesses the systemic importance of large commercial banks from the perspective of network structure, emphasizing the role of banks’ transaction behavior and market participation. Different industries and asset classes are also assessed, suggesting that increased attention should be paid to industry risks and regulatory oversight of bank investments. Finally, stress tests confirm that the improved PageRank algorithm is applicable within the multi-layer financial network, reinforcing the need for prudential supervision of the banking system and revealing that the degree of transaction concentration will affect the systemic importance of financial institutions.

## 1. Introduction

There are a variety of agents and channels of risk propagation involved with financial risk, and the risk is more complex today [1,2]. The study of systemic risk based on complex networks has received widespread attention since the global financial crisis in 2008. Generally, most of the research has been conducted on systemic risk based on a single-layer financial network. However, some studies in recent years have indicated that focusing on a single financial linkage cannot fully reveal systemic risk, and studies across multiple financial markets are needed [1,3,4,5,6]. Some studies have studied the systemic risk of multi-layer financial networks using numerical simulation. Several studies have examined systemic risk across different channels of risk propagation, including Guo et al. [1], Caccioli et al. [7], and Lux [8]. Guo et al. [1] found that systemic risk is mainly a result of asset sales caused by bank deleveraging and bank risk contagion, mainly caused by insolvency. Caccioli et al. [7] found that combining two contagion channels poses a higher systemic risk when comparing interbank lending networks with bank–asset bilateral networks. The bank–firm credit network in Lux [8] was constructed according to the distributional characteristics of banks and firms, and the interbank lending network was combined with it to build a multi-layer model. As discussed in Lux [8], the risk contagion effect is non-linear, and the systemic risk contribution is more significant under the bank–firm credit channel.

Some studies empirically analyze systemic risk under different risk contagion channels and multi-layer financial networks. Poledna et al. [4], as well as Montagna and Kok [9], constructed multi-layer interbank networks, focusing on analyzing the contribution of systemic risk at each layer. In these studies, it was found that considering only a single layer would underestimate the systemic risk. Cao et al. [6], Poledna et al. [10], and Wang and Li [11] all constructed multi-layer financial networks based on the direct connections between interbank transactions and their indirect connections with external assets. They used the DebtRank method to measure systemic risk across different contagion channels. According to these studies, focusing only on direct interbank exposures would significantly underestimate the overall systemic risk. Furthermore, they found that there is a nonlinear effect in the total systemic risk within multi-layer financial networks. In addition, Silva et al. [5] also developed a multi-layer financial network based on interbank lending and bank–firm credit. It is found that topology plays a significant role in contagion and that state-owned banks are most susceptible to real sector shocks. Using China’s actual credit data from 2016, Li et al. [12] constructed a multi-layer bank–firm credit network model. They determined that firms are the main contributors to systemic risk, with large banks and small firms having higher DebtRanks.

Based on a review of relevant studies within multi-layer financial networks, it is found that current studies primarily examine systemic risk within multi-layer financial networks, with less attention given to the issue of systemic importance. The above-mentioned research has contributed significantly to the understanding of financial risk’s origins, propagation paths, and cumulative effects. Following the global financial crisis, countries have strengthened financial regulation, shifting from microprudential to macroprudential regulation [13]. Furthermore, the Basel Committee on Banking Supervision has highlighted the importance of identifying key participants within financial networks for assessing the risk of the complex financial system [14]. At the same time, due to the current process of economic integration and the recovery of economies worldwide, economic participants are increasingly interacting with each other, and financial links have become more complex. For systemic risk management and financial regulation, it is imperative to identify systemically important participants effectively [6,14,15,16,17]. Therefore, this study aims to evaluate systemic importance within multi-layer financial networks.

In assessing the systemic importance of institutions, some studies focus on assessing the contribution of individual institutions to the overall systemic risk within the financial system, such as ΔCoVaR, SES, MES, and SRISK [18,19,20], which have been widely used. They are market-based systemic risk indicators, based on conditional loss probability measurements, but they fail to take into account the network interconnectedness between financial institutions [21]. It is crucial to consider fully the complex interconnectedness among entities in the financial system when identifying systemic importance [16,17]. The studies by Cao et al. [6], Wang and Li [11], Ma et al. [17], and Poledna et al. [22] identified systemically important institutions (banks or firms) using DebtRank. Cao et al. [6] constructed a two-layer financial network based on interbank lending and cross-shareholdings between financial institutions, identifying systemically important financial institutions (SIFIs) within the financial system and confirming that multi-layer networks play a non-linear role in risk propagation. Based on interbank lending and common asset holdings, Wang and Li [11] constructed multi-layer financial networks to identify China’s systemically important banks. Ma et al. [17] and Poledna et al. [22] constructed multi-layer bank–firm financial networks. According to the former, only a small fraction of banks and firms exhibited systemic importance characteristics, and they were highly homogeneous; the latter primarily identified medium-sized firms as systemically important and proposed that systemically important financial institutions (SIFIs) could be extended directly to firms as well. In addition, some studies assess systemic importance based on network centrality. Aldasoro and Alves [14] measured the degree and strength of bank centrality using the PageRank algorithm [23]. Page et al. developed the PageRank algorithm [24], based on the idea that a web page’s page rank is influenced by not just how many links are pointing to it, but also by how central (i.e., the centrality) the page providing the link is. Yun et al. [25] found that PageRank was better at capturing the network structure of financial institutions than CoVaR and MES, thereby aiding in measuring the importance of financial institutions. In order to measure the systemic importance of financial institutions, Huang and Wang [16] constructed a volatility spillover network and developed a comprehensive network centrality index using five types of network centrality (degree centrality, closeness centrality, betweenness centrality, modified Katz centrality, and information centrality).

Methods for assessing systemic importance such as those mentioned above have greatly contributed to this field of research. However, the traditional market-based systemic risk indicators cannot explain the network interconnectedness of financial institutions; DebtRank considers the network interconnectedness of entities, but is generally applied to single-layer or segmented interbank lending networks or bank–firm credit networks. The multi-layer financial network of this paper has diverse agents, as opposed to a single economic entity, resulting in more complex financial connections. The PageRank algorithm is capable of better capturing the characteristics of a network, and this paper relies on the PageRank algorithm to assess the systemic importance of agents within multi-layer financial networks. 

Further, it is found that there is relatively little research on the assessment of systemic importance within China’s multi-layer financial networks. On the one hand, the global economic growth rate has been declining in recent years, and the European and American banking crises last year also exacerbated financial turmoil and spread of financial risks. On the other hand, the domestic consumption of China has decreased, and the economic growth has slowed. Furthermore, since 2023, China’s real estate has been facing deflationary pressure caused by overcapacity and insufficient consumption, with real estate falling as a whole and real estate risks gradually exposed. China’s financial system has been affected by both domestic and international risk shocks. Financial stability has always been a priority for the Chinese government. During the 2023 Chinese Two Sessions, the National Financial Supervisory Administration was announced, to improve financial risk identification, early warning, and prevention. Implementing macroprudential supervision effectively and preventing financial risks precisely, with the identification of systemically important institutions being a key component [14]. Therefore, this paper focuses on the assessment of systemic importance within China’s multi-layer financial networks.

The closest works to this paper are those of Cao et al. [6], Aldasoro and Alves [14], and Ma et al. [17]. Firstly, these three studies focus primarily on further decomposition based on a single financial connection when describing complex financial networks. Using interbank lending and cross-shareholding among financial institutions, Cao et al. [6] constructed a two-layer financial network; Aldasoro and Alves [14] decomposed the interbank network according to maturity dates and instruments; Ma et al. [17] decomposed the bank–firm network based on loan terms and investment cycles. The DebtRank method was used by all three to assess institutions’ systemic importance. Nevertheless, this paper examines three agents—banks, firms, and assets—and the complex financial connections between them are thoroughly explained, identifying various connections such as interbank lending, bank–firm credit, and banks holding portfolios, in order to characterize the financial network. This paper proposes a multi-layer financial network that differs from theirs significantly. Because the financial network of this paper involves various connections, which makes it more complex, the PageRank algorithm is more appropriate for capturing its network characteristics. This paper uses the PageRank algorithm to assess the systemic importance of financial institutions in order to demonstrate the critical role played by multi-layer financial network structures. Based on the original PageRank algorithm, this paper extends its application to multi-layer financial networks by considering the weight of financial transactions. Also, when analyzing the systemic importance of institutions, this paper not only identifies systemically important banks, but also identifies industry risks and risky assets in comparison to the three existing studies, providing relevant recommendations for regulators as well as the banking industry.

This paper collects the actual data of the Chinese banking system and listed firms in 2021. Firstly, this paper estimates China’s multi-layer financial network system by estimating the interbank lending network, the bank–firm credit network, and the bank–asset portfolio network based on the multi-layer financial network model proposed by Gao [26]. Based on the statistical data of China’s multi-layer financial networks, this paper also analyzes the characteristics of single-layer networks and multi-layer financial networks. An improved PageRank algorithm is then proposed to identify China’s systemically important institutions. This paper also conducts a stress test to further verify the applicability of the improved PageRank algorithm within multi-layer financial networks. The possible contributions of this paper are:

1. In previous research on multi-layer financial networks, fewer inter-agent linkages were examined, and multiple agents and the complexity of their links were not fully considered. Taking into account various financial linkages, including interbank lending, bank–firm credit, and bank-holding portfolios, this paper estimates China’s multi-layer financial network based on the complete balance sheets of banks. It is difficult to conduct comprehensive credit network estimations based on the balance sheets of the banking system due to the limited availability of data (banks rarely provide complete credit transaction data). This paper proposes an improved estimation method that estimates the credit characteristics of a class of firms by the credit characteristics of some firms. The total loan data from bank balance sheets and loan information from announcements of listed firms are utilized to estimate the complete bank–firm credit network and eventually obtain the complete multi-layer financial network.

As a result of such an estimation method, Chinese multi-layer financial networks have relatively small estimation errors, allowing this study to analyze China’s single-layer and multi-layer financial networks comprehensively, as well as determine the actual network characteristics of the Chinese banking system and establish a basis for systemic importance evaluation.

2. It is important to note that the original PageRank algorithm focuses only on the linkages between agents. Based on the PageRank index for a single-layer network, this paper considers the impact of agents’ transaction amounts on systemic importance and proposes the PageRank index for the multi-layer financial network.

In contrast to traditional methods for assessing systemic importance, this study employs an improved PageRank method based on network structure centrality. By taking this approach, we are able to not only evaluate the systemic importance of banks but also assess systemic importance across different industries and asset classes. Additionally, it provides a reference for banks’ investment decisions and clarifies the regulatory authority’s direction on risk monitoring.

3. In addition to empirical research, this paper conducts stress tests on China’s multi-layer financial network. The results of the stress tests confirm the applicability of the improved PageRank algorithm proposed in this paper for assessing systemic importance within multi-layer financial networks. By combining empirical analysis with stress test results, this paper further explains the intrinsic mechanisms behind ‘too big to fail’ and ‘too central to fail’ for systemically important banks. It emphasizes the necessity of prudential supervision for the banking system and provides guidelines for managing systemic risks by the relevant regulatory authorities.

In summary, this study presents a new network estimation method for empirical studies within multi-layer financial networks and a new perspective and assessment methodology for studying systemic importance. As another example of ‘Too central to fail,’ this study also supports ‘Too big to fail’. It provides a reference for the current efforts to mitigate and prevent systemic risks.

## 2. Methodology for Assessing the Systemic Importance of Multi-Layer Financial Net-Works

### 2.1. Estimation of Multi-Layer Financial Networks

This paper estimates the bank–firm credit network, the bank–asset portfolio network, and the interbank lending network based on processing bank balance sheets and loan data from listed firms.

In the multi-layer financial network system of this paper, the assets of bank i mainly include loans issued ($L_{i}$), financial assets held ($F_{i}$), interbank lending funds ($LO_{i}$), cash assets ($C_{i}$) and other assets ($OA_{i}$) (non-financial assets such as fixed assets and intangible assets); the liabilities of bank i mainly include interbank borrowing funds ($LI_{i}$) and other liabilities ($OL_{i}$) (savings, etc.); and the net equity of bank i is $E_{i}$, thus, the balance sheet of bank i can be expressed as:

(1) $$L_{i}+F_{i}+LO_{i}+C_{i}+OA_{i}=LI_{i}+OL_{i}+E_{i}$$

Due to the unavailability of data on all stock credit transactions between banks and firms, most previous studies have directly used the publicly available data on credit transactions between banks and firms that can be collected (from some database or regulatory authority) to construct the bank–firm credit network directly. It does not include all of the bank’s credit transactions on its balance sheet, but only a portion of them. As in the work of Silva et al. [5], Li et al. [12], Poledna et al. [22], and Luu and Lux [27], they mainly focus on bank–firm credit risk. Three types of financial transactions are examined in this study: credit between banks and firms, bank investments, and interbank lending. By using the aforementioned method, it is impossible to estimate the bank’s bank–firm credit adequately, resulting in errors in the proportion of the bank’s transactions in various financial markets. In order to ensure that the complete credit data in the balance sheet of banks can be estimated, and the proportion of banks’ transactions in each financial market is reflected as realistically as possible. An improved estimation method is proposed in this paper. Using the credit characteristics of a portion of firms, it estimates the credit characteristics of a class of firms, estimating and obtaining a complete bank–firm credit network using total loan data from banks’ balance sheets and loan information from listed firms’ announcements. If the number of banks with collected balance sheets is denoted as $N_{B}$, and the number of listed firms with disclosed loan transactions is $N_{F}$, then the disclosed bank–firm credit linkages can be represented by matrix $V^{bf}=v_{i,j}^{bf}(N_{B}\times N_{F})$. The element $v_{i,j}^{bf}$ within the matrix indicates the loan amount announced by listed firm j to bank i. Therefore, in the estimated bank–firm credit network of the multi-layer financial system, the number of banks and firms are $N_{B},N_{F}$, respectively. The amount of loans issued by bank i to firm j can be expressed as follows:

(2) $$a_{i,j}^{bf}=L_{i}\frac{v_{i,j}^{bf}}{\sum_{j=1}^{N_{F}}v_{i,j}^{bf}}$$

The total loans granted by bank i is $L_{i}=\sum_{j=1}^{N_{F}}a_{i,j}^{bf}$, firm j in the estimated bank–firm credit network in this paper represents a collection of firms with the same debt characteristics.

Bank balance sheets contain information about the various types of financial assets held by banks. Using this information, this paper identifies the bank–asset portfolio network. As there are some differences in the financial assets held by each bank, if all the banks in the system hold $N_{A}$ types of financial assets, and the amount of financial asset m held by bank i is $a_{i,m}^{ba}$, then the total amount of financial assets held by the bank is $F_{i}=\sum_{m=1}^{N_{A}}a_{i,m}^{ba}$.

It is usually only possible to obtain the total interbank lending and interbank borrowing from the bank’s balance sheet. Referring to Lux [8] and Gao [26], this paper constructs an interbank lending linkage matrix based on a probability function related to the bank’s assets. According to this paper, the probability of an interbank lending relationship between bank i and bank k is as follows:

(3) $$p_{i,k}=d{\left(\frac{A_{i}}{A_{\max}}\right)}^{a_{1}}{\left(\frac{A_{k}}{A_{\max}}\right)}^{a_{2}}$$

where $A,A_{\max}$ represent the total assets of the bank and the maximum assets of the system, respectively, and $d,a_{1},a_{2}$ are constant parameters. The total amount of interbank lending funds of bank i is $LO_{i}=\sum_{k=1}^{N_{B}}a_{i,k}^{bb}$, where $a_{i,k}^{bb}$ is the amount lent by bank i to bank k, then:

(4) $$a_{i,k}^{bb}=LO_{i}\frac{p_{i,k}A_{k}}{\sum_{h=1}^{N_{B}}p_{i,h}A_{h}}$$

### 2.2. PageRank in a Multi-Layer Financial Network

Based on Yun et al. [25], this paper uses PageRank to measure the systemic importance of banks in multi-layer financial networks. Yun et al. [25] used Granger causality networks to create the effect matrix, but they did not use interbank transaction data, which led to errors in estimating the influence between banks. This paper estimates the financial network based on interbank transaction data, and fully considers the influence of interbank transaction amount on the influence between banks when portraying interbank linkages, so that interbank linkages are presented as weighted directed linkages. If the influence index of bank i to bank k is $E_{i,k}$, the PageRank index of bank i is:

(5) $$PR_{i}=\frac{1-\alpha }{N_{B}}+\alpha \sum \left(PR_{k}E_{k,i}\right)$$

where α is the damping factor, usually set to 0.85.

There is no direct linkage between banks in the bank–firm credit network, but indirect linkages are established between banks through the common debtor, which allows a bank to transmit its risk to other banks. Through the debt relationship between listed firm j and its creditor banks i, k, it is possible to calculate the influence of this creditor bank i on another creditor bank k. Suppose the influence of bank i on bank k is $e_{i,k}^{bf}$ in the bank–firm credit network, then:

(6) $$\left\{\begin{array}{l} e_{i,k}^{bf}=\sum a_{i,j}^{bf}\frac{a_{k,j}^{bf}}{\sum a_{k,j}^{bf}},\;(a_{i,j}^{bf}>0,a_{k,j}^{bf}>0,k\neq i)\\ E_{i,k}^{bf}=\frac{e_{i,k}^{bf}}{\sum e_{i,k}^{bf}},\;\;\;(e_{i,k}^{bf}>0) \end{array}\right.$$

$E_{i,k}^{bf}$ represents the normalized value of the influence of bank i on bank k in the bank–firm credit network, i.e., the influence index of bank i on bank k.

Similarly to the bank–firm credit network, the bank–asset portfolio network establishes indirect links between banks by holding common assets. If a bank has to sell its assets, the other banks that hold the assets will also suffer asset losses. Currently, the influence of bank i on bank k can be calculated based on the value of their common assets. Suppose the influence of bank i on bank k is $e_{i,k}^{ba}$ in the bank–asset portfolio network, then:

(7) $$\left\{\begin{array}{l} e_{i,k}^{ba}=\sum a_{i,m}^{ba}\frac{a_{k,m}^{ba}}{\sum a_{k,m}^{ba}},\;(a_{i,m}^{ba}>0,a_{k,m}^{ba}>0,k\neq i)\\ E_{i,k}^{ba}=\frac{e_{i,k}^{ba}}{\sum e_{i,k}^{ba}},\;\;\;\;\;(e_{i,k}^{ba}>0) \end{array}\right.$$

$E_{i,k}^{ba}$ represents the normalized value of the influence of bank i on bank k in the bank–asset portfolio network, i.e., the influence index of bank i on bank k.

A direct link between banks is established based on the amount of lending and borrowing in an interbank lending network, which makes it possible to measure the influence of banks directly by lending and borrowing. Suppose the influence of bank i on bank k is $e_{i,k}^{bb}$ in the interbank lending network, then:

(8) $$\left\{\begin{array}{l} e_{i,k}^{bb}=a_{i,k}^{bb},\;\;(a_{i,k}^{bb}>0,k\neq i)\\ E_{i,k}^{bb}=\frac{e_{i,k}^{bb}}{\sum e_{i,k}^{bb}},\;\;\;\;\;(e_{i,k}^{bb}>0) \end{array}\right.$$

$E_{i,k}^{bb}$ represents the normalized value of the influence of bank i on bank k in the interbank lending network, i.e., the influence index of bank i on bank k.

Using the above influence indexes among banks in the bank–firm credit network, the bank–asset portfolio network, and the interbank lending network, the PageRank index ($PR_{i}^{bf},PR_{i}^{ba},PR_{i}^{bb}$) for bank i in each network are calculated by applying the PageRank algorithm, i.e., the systemic importance indexes. The PageRank index of bank i can be calculated as follows under a multi-layer financial network:

(9) $$PR_{i}=\frac{PR_{i}^{bf}\sum a_{i,j}^{bf}+PR_{i}^{ba}\sum a_{i,m}^{ba}+PR_{i}^{bb}\sum a_{i,k}^{bb}}{\sum a_{i,j}^{bf}+\sum a_{i,m}^{ba}+\sum a_{i,k}^{bb}}$$

### 2.3. Stress Test of Multi-Layer Financial Networks

This paper also performs stress tests on the multi-layer financial network described above, where the failure of one bank could cause failure of other banks or even trigger a system-wide failure if the failure spreads throughout the network. If bank i fails at time step t, its liquidation will begin with the sale of its financial assets and the recovery of loans and interbank borrowings it has extended to repay its debts.

In the multi-layer financial network system, banks’ sale of financial assets leads to asset depreciation, further spreading risk. According to Caccioli et al. [28], this paper introduces a market impact function to measure fire-sales effects. As a result of the bankruptcy of bank i, the depreciation of assets at time step t can be expressed as follows:

(10) $$\left\{\begin{array}{l} f(m,x_{m,t})=e^{-\sigma x_{m,t}}\\ x_{m,t}=\frac{a_{i,m,t}^{ba}}{\sum a_{i,m,t}^{ba}}\\ a_{k,m,t}^{ba}=a_{k,m,t-1}^{ba}f(m,x_{m,t}) \end{array}\right.$$

$x_{m,t}$ is calculated as the ratio of the value of asset m sold by failed bank i versus the sum value of asset m in the system, and σ represents asset price sensitivity, i.e., the fluctuation in the value of asset m due to fire sales. Because of the failure of bank i, bank k suffered an asset loss of $\sum (a_{k,m,t-1}^{ba}-a_{k,m,t-1}^{ba})$.

Liquidation of insolvent bank loans will result in credit losses suffered by the firms, a decrease in its access to loans, liquidity problems, and ultimately, bankruptcy. The creditor banks will also suffer financial losses when a firm defaults on its credit. Following the insolvency of bank i, the rate of access to loans for its debtor firms j at time step t is as follows:

(11) $$\varphi_{j,t}=\frac{\sum a_{i,j,t-1}^{bf}-a_{i,j,t-1}^{bf}}{\sum a_{i,j,0}^{bf}}$$

The firm defaults on its credit if $\varphi_{j,t}<\varphi_{0}$. $\varphi_{0}$ is the minimum rate of access to loans a firm needs to guarantee its production operations. When firm j defaults on its credit, its creditor bank k suffers financial loss of $\sum a_{k,j,t-1}^{bf}$.

Likewise, the insolvent bank’s interbank lending funds will be liquidated, depositors’ rights and interests will be protected, and its creditor banks may suffer capital losses. Upon insolvency of bank i, the capital loss of its creditor bank k can be denoted as $-E_{i,t}\frac{a_{k,i,t-1}^{bb}}{L_{i,t-1}}$, at which point the net equity of the insolvent bank meets $E_{i,t}\leq 0$.

If the failure of bank i leads to the failure of other banks, the risk will continue to propagate until no new bank fails or all banks in the system fail.

## 3. Results

### 3.1. Data

Using the CSMAR database, this paper collects bank balance sheet data and announced loan data for Chinese listed firms in 2021. Invalid loan data, such as syndicated loans, loans from non-bank financial institutions, and loans without specific banks, were excluded from listed firms’ loan data. Following data cleaning, 9640 pieces of bank–firm credit data were obtained from 391 banks and 1058 listed companies, addressing issues such as non-standard bank names. A total of 8234 bank and firm loan data, including 149 banks and 1029 firms located in all 19 industry classifications of listed firms, were screened due to the availability of bank balance sheet data.

Based on the bank balance sheets, direct access can be obtained to loans, cash, and interbank borrowing funds. The balance sheet accounts were sorted to determine 12 categories of financial assets held by banks, namely precious metals, derivative financial assets, reverse repurchase agreements, receivables, available-for-sale financial assets, trading financial assets, bonds, other debt investments, equity investments, held-to-maturity investments, long-term equity investments, and investment properties. Additionally, other categories of assets, such as fixed assets and intangible assets, are harmonized and classified as other assets, as they account for a small percentage of total assets. This study examined 149 banks, including six large state-owned commercial banks, three policy banks, 11 joint-stock commercial banks, 68 city commercial banks, 39 rural commercial banks, two rural banks, and 20 foreign banks.

### 3.2. Multi-Layer Financial Network of the Chinese Banking System

**Figure 1:**
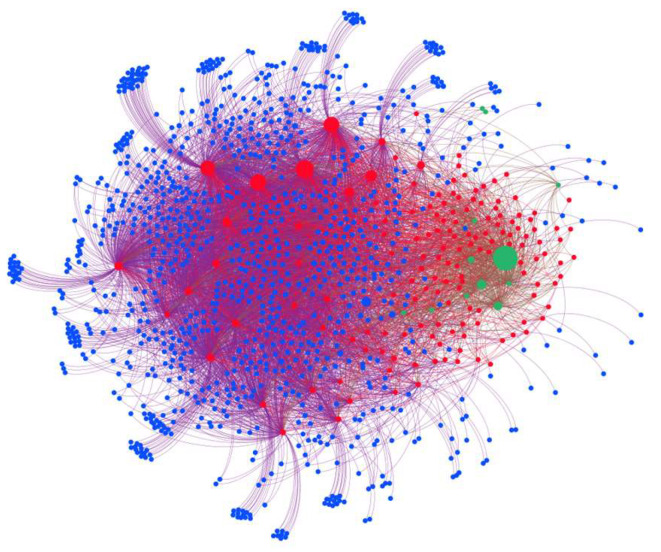
Multi-layer financial network of the Chinese banking system. (Red: banks; green: financial assets; blue: firms).

Based on the estimation method presented in Section 2.1 and using the collected balance sheet data of China’s banking system and loan data of listed firms, a multi-layer financial network of the Chinese banking system has been estimated, as shown in Figure 1. Figure 1 shows that banks are central nodes in the multi-layer financial network, and large banks clearly have more financial connections. It is evident that financial investments are polarized, with a few types of financial assets being significantly larger than others. Furthermore, firm nodes are characterized by significant local aggregation.

**Figure 2:**
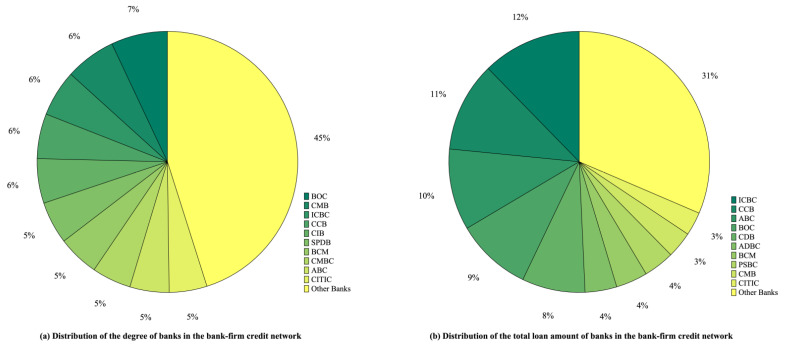
Distribution of degree and total loan amount of banks in the bank–firm credit network. (BOC: Bank of China; CMB: China Merchants Bank; ICBC: Industrial and Commercial Bank of China; CCB: China Construction Bank; CIB: Industrial Bank; SPDB: Shanghai Pudong Development Bank; BCM: Bank of Communications; CMBC: China Minsheng Bank; ABC: Agricultural Bank of China; CITIC: China CITIC Bank; CDB: China Development Bank; ADBC: Agricultural Development Bank of China; PSBC: Postal Savings Bank of China).

A statistical analysis was performed to explore the network characteristics of the multi-layer financial network of China’s banking system. The results are shown in Table 1. Based on Table 1, the network density of the multi-layer financial network and each single-layer network of the Chinese banking system is low, indicating a sparse network characteristic. The average clustering coefficients of the three single-layer networks are high, and the single-layer networks display typical characteristics of local aggregation. In addition, further analysis shows that the standard deviations of the in-degree and out-degree are large in all three single-layer networks, suggesting a significant heterogeneity in the distribution of transactions among financial institutions in the market for bank–firm credit, bank investment, and interbank lending. Furthermore, all three networks have relatively high in- and out-degree weighted power rate indexes. The bank–firm credit network has both high in-degree and out-degree weight power rate index, yet relatively few banks issue a large number of loans, and the loans are concentrated in a small number of firms. It can be seen in Figure 2 that ten large commercial banks account for 55% of all credit linkages and 69% of all credit transactions. Clearly, bank–firm credit business is correlated with asset size, and credit services are severely polarized. Similar results were found by De Masi and Gallegati [29]. Despite the fact that a small number of firms have easier access to financing, a large number face difficulties with financing, and regulators should provide policy guidance on firm financing. Furthermore, banks need to manage credit risks effectively in order to avoid risks arising from over-concentration of credit in some firms.

**Figure 3:**
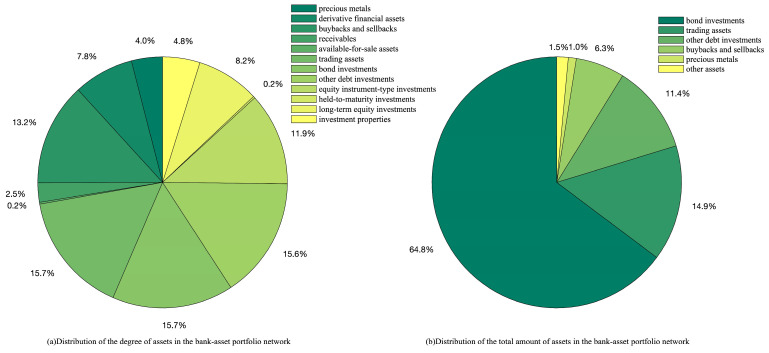
Distribution of the degree of asset classes and the total amount of assets in the bank–asset portfolio network.

According to Figure 3, banks’ investments are concentrated in a few asset classes in the bank–asset portfolio network. Among them, the number of banks investing in bond assets is ranked first, along with the total amount of their investments, where the total amount of banks’ investments in bond assets accounts for 65% of their total investment. As shown by the data, banks currently have highly similar investment strategies, where the impact of fluctuations in the value of a single asset class can be magnified, makes it critical for banks to manage and respond to investment risk. To prevent systemic risks and contagion, banks and regulatory authorities must conduct post-investment risk management. Alternatively, banks should seek breakthroughs in asset classes with significant investment selection differences (such as precious metals, derivative financial assets, and equity instruments) to maximize investment returns.

**Figure 4:**
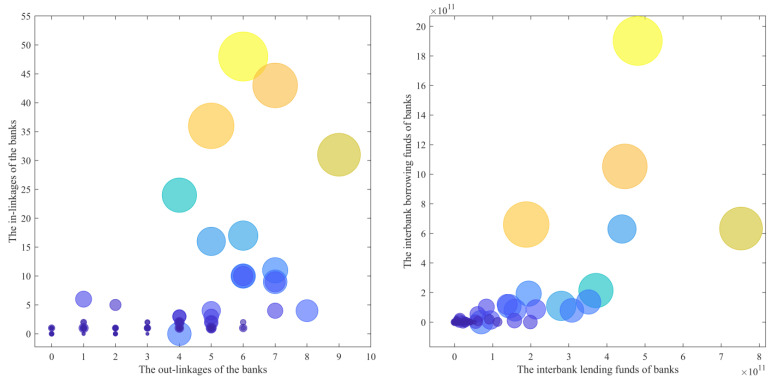
Degree of banks and total lending amount in the interbank lending network.

In the interbank lending network, the in-degree weighted power index is significantly higher than the out-degree weighted power index, which indicates that a small number of banks hold a large amount of interbank debt. When combined with Figure 4, it is clear that there is little difference between the out-linkages of each bank in the interbank lending market, while the in-linkages have a significant gap; this is even more evident when it comes to the amount of interbank lending and borrowing. Several large banks accounted for most of the interbank lending funds, and large banks were at the forefront of the interbank lending market, both in terms of interbank lending and interbank borrowing funds, due to their large asset sizes and high liquidity requirements. Based on the analysis, large commercial banks serve as credit intermediaries in the financial market. As a result of their risk resistance capabilities and the ability to provide increased interbank funding to small and medium-sized banks, they contribute significantly to maintaining financial stability and alleviating liquidity risks. With the current global economic downturn and increased demand for financing, regulatory authorities should conduct a rational analysis of the interbank lending market’s transaction conditions, adjust the money supply accordingly, and keep liquidity risks at bay while avoiding insufficient market supply.

Overall, the multi-layer financial network of China’s banking system exhibits distinct sparse network characteristics. The distribution of transactions between financial institutions in bank–firm credit, bank investments, and interbank lending markets is clearly uneven. In response to these uneven phenomena, on the one hand, the regulatory authorities should manage the relevant financial risks while regulating the money supply, solving the financing problems of firms, and contributing to the recovery of the economy through policy guidance and stronger supervision. On the other hand, the banking industry should strengthen differentiated competition in investment choices, manage risk effectively, and maximize profits.

### 3.3. Systemic Importance within China’s Multi-Layer Financial Network

Based on the PageRank index, the top ten systemically important banks in China’s banking system are presented in Table 2. The results indicate that large state-owned commercial banks play a significant role in China’s banking system. Industrial and Commercial Bank of China (ICBC) ranks first in single-layer and multi-layer financial networks, consistent with its position as the world’s largest bank. Agricultural Bank of China (ABC), China Construction Bank (CCB), Bank of China (BOC), Bank of Communications (BCM), and Postal Savings Bank of China (PSBC) also ranked relatively stable in terms of importance. In addition, China Merchants Bank (CMB), Industrial Bank (CIB), and China CITIC Bank (CITIC) are ranked in the top ten. Shanghai Pudong Development Bank (SPDB), the Export-Import Bank of China (CEXIM), and China Minsheng Bank (CMBC) exhibit higher PageRank index in the bank–firm credit network and interbank lending network, respectively. As a result, these banks are more prominent within a single financial market, but their influence on the overall financial system is not as significant yet.

**Figure 5:**
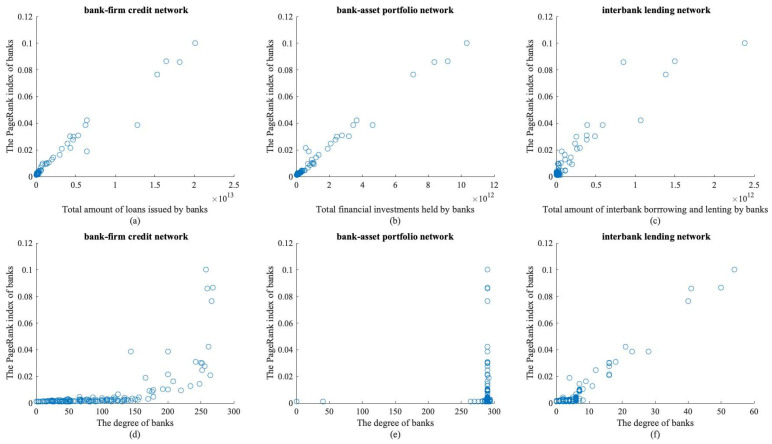
Degree, the amount of transactions and the PageRank index of the bank in each financial market.

Combined with Figure 5, it is evident that the transaction amount held by each bank in the bank–firm credit network, bank–asset portfolio network, and interbank lending network has a positive linear relationship with its PageRank index. The degree of most banks in the bank–firm credit network is relatively small, and so is their PageRank index, while a small number of banks have higher degrees and higher PageRank indexes. The degree of banks in the bank–asset portfolio network is relatively concentrated, most banks have lower PageRank indexes, and a small number of banks have higher PageRank indexes. The degree of banks is close to a positively correlated linear relationship with their PageRank indexes in the interbank lending network, but some banks have significantly higher degrees than others. Therefore, this paper argues that in a single financial market, the greater the total amount of trading funds held by a bank and the more trading relationships it has, the higher its PageRank index and the more important it is in the banking system. When a risk occurs, the greater the total amount traded, the greater the potential loss of counterparty funds, resulting in cascading counterparty defaults and, therefore, risk contagion. With more trading relationships, the bank has a broader range of channels through which it can spread its risk, resulting in higher cumulative risks. It is worth noting that the degree of different banks is highly concentrated in the bank–asset portfolio network, indicating a high level of asset overlap between banks. Additionally, the degree of some banks in the bank–firm credit network and the interbank lending network is significantly higher than that of other banks, which, coupled with the asset size of the banks, suggests that large banks dominate both networks. Due to their substantial scale, large commercial banks can attract a concentration of trading counterparts and provide a more significant amount of trading capital to the financial markets. Simultaneously, they play the role of credit intermediaries in the interbank market, promoting market equilibrium and thus contributing to financial stability.

The above study confirms the systemic importance of large commercial banks in the financial system from the perspective of network structure, and the weighted centrality of banks is closely related to their systemic importance. This proves that “Too central to fail” and “Too big to fail” are not in conflict and that a bank’s transaction behavior and market participation level are the key determinants. China’s systemically important banks have large asset sizes and play a weathervane role in the financial market. Identifying and strengthening the regulation of systemically important banks helps to improve the transmission mechanism of monetary policy, promote fair and orderly market competition, and enhance the robustness of the banking system, effectively preventing and resolving systemic financial risks.

By analyzing the common creditor banks of different firms and the common investment banks of different asset classes within the bank–firm credit and bank–asset portfolio networks, this paper calculates the influence index among different firms and the influence index between different asset classes. Based on this, PageRank indexes for different industries and asset classes were calculated. The industries with the top five PageRank indexes are shown in Table 3. PageRank indexes for the manufacturing, wholesale and retail trade, and real estate industries rank among the top three, indicating that these three industries have the highest risk coefficients. When they are at risk, they have a more significant impact on other industries and are more likely to trigger systemic risks. The manufacturing industry is a critical component of the country’s economic development and a symbol of the country’s industrialization level. Based on further analysis of the data, it can be seen that the number of firms in the manufacturing industry amounts to 673, accounting for over 65% of the total; the cumulative risk is, therefore, higher, and its PageRank index reaches 0.6383. China’s wholesale and retail industry is one of the most market-oriented and competitive; the real estate industry, on the other hand, is naturally characterized by fundamentals and risks and has significant long-term capital requirements. The wholesale and retail trade and real estate industries have significantly higher average PageRank indexes than other industries, indicating that the risk coefficients of single firms are larger in these industries and should pay more attention to industry risk. Particularly since the global financial crisis in 2008, countries have intensified risk management in areas such as real estate, working to deflate property bubbles and strictly guard against systemic risks. Regulatory authorities need to enhance risk monitoring to prevent the aggregation of industry risks. Banks, on their part, must strengthen loan approval and management in critical industries, exercising strict supervision over the use of funds.

The assets with the top five PageRank indexes are shown in Table 4. Table 4 shows that banks’ bond investments and trading financial assets have a much higher PageRank index than other assets. Due to the high overlap in investment portfolios among banks, the total amount of these assets significantly affects the PageRank index, with the total amount of these two types of assets alone accounting for 79.8% of all bank financial assets. Therefore, banks and regulators should strictly monitor banks’ investment behavior, guiding banks to engage in diversified investments to prevent large amounts of capital aggregation and systemic risks arising from defaults on single assets.

### 3.4. Stress Test of China’s Multi-Layer Financial Network

In addition to the above empirical studies, this paper conducts a stress test for the Chinese banking system under the multi-layer financial network. This paper sets a minimum rate of access to loans $\varphi_{0}$ for firms at 0.8. 149 banks are chosen sequentially as the initial failing banks to determine the stability of the entire banking system when various banks’ bankruptcy risks occur. According to the stress test, the top ten banks with the highest default probability (bank bankruptcy or capital adequacy ratio falls below regulatory requirements) triggered by an initial bank default are presented in Table 5, essentially the same as the top ten banks ranked by PageRank in Table 1. This also confirms the applicability of the improved PageRank algorithm proposed in this paper within the multi-layer financial network. Based on Table 5, the failure of the Industrial and Commercial Bank of China (ICBC) and the Agricultural Bank of China (ABC) resulted in the default of all banks in the banking system. The failure of the China Construction Bank (CCB), Bank of China (BOC), and China Development Bank (CDB) also led to the default of almost all banks (only one survives). This illustrates once again the importance of state-owned large commercial banks to the Chinese banking system and their influence over others.

The analysis of the multi-layer financial network of China’s banking system in Section 3.2 shows that large banks tend to own more debtor firms. Once the bank operates poorly and encounters risks, it will make a large number of firms illiquid as it loses credit funding sources, resulting in bankruptcy. Bankruptcies of these firms would cause losses to other creditor banks, thereby spreading risks throughout the entire banking system. Meanwhile, large banks are well capitalized and hold a large amount of financial assets. Liquidation of a large bank will lead to dramatic fluctuations in the price of these financial assets, resulting in losses for other banks that hold the same assets. Additionally, large banks act as credit intermediaries, facilitating the transfer of funds between them. However, the bankruptcy or liquidation of a large bank would prevent interbank capital flows, resulting in a liquidity risk. Therefore, large commercial banks are of significant systemic importance in China’s banking system.

**Figure 6:**
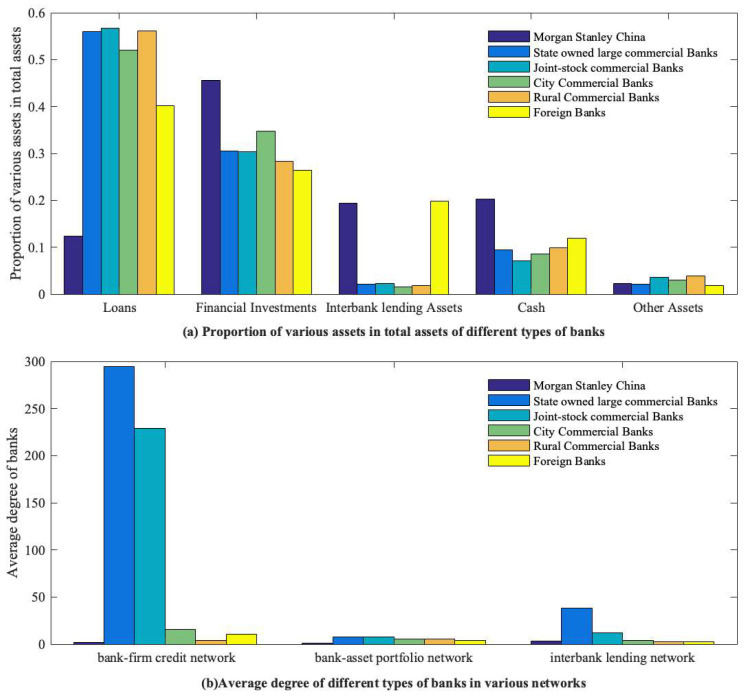
The proportion of each asset in total assets and the average degree in various networks of different types of banks in various networks.

It should be noted that Morgan Stanley China survived the risks of contagion from the China Construction Bank (CCB), Bank of China (BOC), and China Development Bank (CDB). As shown in Figure 6, Morgan Stanley China holds a much lower proportion of riskier credit assets than the average for all types of banks and a much higher proportion of lower-risk lending and risk-free cash assets. Meanwhile, the degree of the bank in each network is also lower than the average across all bank types. Additionally, through comparative analysis of the types of assets held by different banks, it has been observed that foreign banks generally hold a smaller proportion of high-risk assets and a larger proportion of low-risk assets. This also indicates that the business and investment activities of foreign banks in China are more prudent.

The analysis suggests that the co-debtor channel is the most important channel for risk propagation within the banking system, and that the risk of debt default is also the most significant risk that the banking system faces. Among the foreign banks mentioned above, the asset distribution of Morgan Stanley Bank shows a discrepancy from the industry average, with its actual risk exposure being significantly lower than the average risk level of the banking sector. So, it is less affected in the process of risk propagation and can maintain a higher level of risk resistance, avoiding bankruptcy. Foreign banks have more concerns about the market environment and regulatory policies due to their non-domestic operations and, therefore, adopt more prudent business strategies. The results of stress tests still indicate that prudential supervision of the banking system and guiding it towards more rational business activities are crucial measures for managing systemic risks.

In addition, only the probability of a systemic default caused by the failure of the Bank of Communications did not rank among the top ten, ranking 12th. Moreover, further analysis showed that the Bank of Communications (BCM) and Postal Savings Bank of China (PSBC) triggered significantly fewer bank failures than the other four large state-owned commercial banks under the direct impact of bank failures (as shown in Table 6). After some banks absorb risks, their ability to resist risks decreases, and they are subject to strict regulatory restrictions (with capital adequacy ratios lower than regulatory requirements). However, they also block the spread of risks and do not directly transmit them, resulting in a smaller overall system risk.

**Figure 7:**
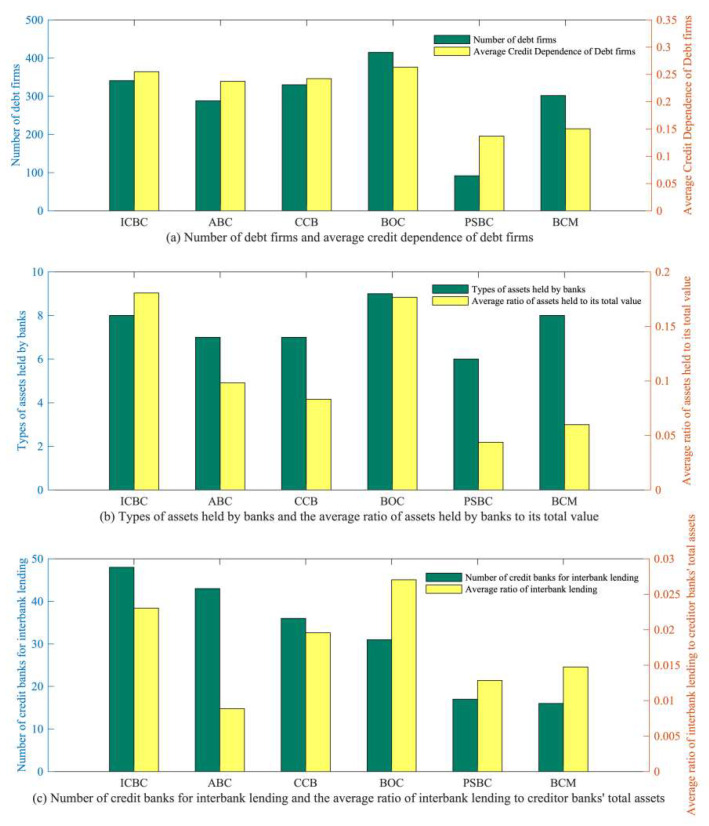
Counterparties of the six largest state-owned banks across trading markets. (ICBC: Industrial and Commercial Bank of China; ABC: Agricultural Bank of China; CCB: China Construction Bank; BOC: Bank of China; PSBC: Postal Savings Bank of China; BCM: Bank of Communications).

Based on Figure 7, it is clear that the Postal Savings Bank of China (PSBC) owns the least number of debt firms and that its debt firms have the lowest credit dependence on the bank in the bank–firm credit network. Although the Bank of Communications (BCM) has more debtor firms, those debt firms are also less dependent on the bank for credit. The remaining four large state-owned commercial banks have more debtor firms, and their debtor firms have a higher average degree of credit dependence on the bank. With a minimum access rate of 0.8 for firm loans, a credit dependence of over 20% means that when a bank experiences risk, the firm will immediately collapse due to the financial risk caused by loan losses, which will spread rapidly. Thus, the lower the debt firm’s dependence on bank credit, the less likely it is to go bankrupt due to bank risks. Consequently, the scope of risk propagation is smaller, and it is less likely to trigger a cascade of bankruptcies. There is little difference between the types of assets held by the six state-owned banks in the bank–asset portfolio network. However, the proportion of assets held by Postal Savings Bank of China (PSBC) and Bank of Communications (BCM) to their total assets is the lowest. Thus, when they encounter risks, the risk of asset depreciation is also the lowest, and the losses caused to other banks are also smaller. Postal Savings Bank of China (PSBC) and Bank of Communications (BCM) have the smallest number of lending creditor banks in the interbank lending network, and the proportion of lending funds to their creditor banks’ total assets is relatively low. Therefore, when banks encounter risks, they have little impact on their creditor banks. Overall, the Postal Savings Bank of China (PSBC) and Bank of Communications (BCM) are relatively independent of their counterparties in various financial markets among the six major state-owned banks. Therefore, the direct risk caused by their occurrence of risks is also relatively small, and the contagion risk they transmit to other banks through their counterparties is also relatively small, so they have not caused many direct bankruptcies. Additionally, when the Postal Savings Bank of China (PSBC) encounters risks, its number of restricted banks is higher than that of the Bank of Communications (BCM), and its risk propagation cycle is longer. As a result, in the process of risk propagation, these restricted banks are more prone to failures due to the accumulation of risks they face, and ultimately, the number of bank failures caused by the Postal Savings Bank of China (PSBC) is higher than that of Bank of Communications (BCM).

It is argued in this article that when a bank incurs risks, the excessive dependence of its counterparty on the bank can result in the counterparty suffering excessive losses and subsequently going bankrupt. As a result, the risk is spread throughout the entire banking system due to its trading activities with other banks. Therefore, banks and the relevant supervisory authorities should pay attention to counterparty risk, and strengthen centralized review before transactions, to avoid systemic risk arising from the excessive dependence of a single counterparty on a bank and the over-concentration of risk exposure. At the same time, it enhances the supervision of banks with excessively concentrated transactions, restricts their trading activities, reduces risk exposure, and prevents the occurrence of risks.

## 4. Conclusions

Based on data collected from 149 banks and listed companies in China in 2021, this paper estimated the interbank lending network, bank–firm credit network, and bank–asset portfolio network, as well as the multi-layer financial network of the Chinese banking system. First, the network characteristics of the three single-layer and multi-layer financial networks within the banking system were examined based on the relevant indicators of network structure. Using the improved PageRank algorithm, the systemically important institutions in China were identified from a multi-layer network perspective, and further analysis was performed on the systemically important institutions in different industries and asset classes. Finally, a stress test was conducted on China’s multi-layer financial network system.

It has been found that each single-layer and multi-layer financial network in the Chinese banking system exhibits network sparsity, whereas each single-layer network exhibits characteristics of local aggregation. The distribution of transactions among financial institutions is also uneven. Specifically, a relatively small number of banks issue a large amount of loans, while loans are concentrated in a small number of firms in the bank–firm credit network. In the bank–asset portfolio network, banks’ investments are concentrated in a few asset classes, with bond assets holding the largest share. In the interbank lending network, a small number of banks hold significant amounts of interbank debt, and large banks dominate the interbank lending market in terms of interbank borrowings and lending. In response to these uneven phenomena, on the one hand, the regulatory authorities should manage the relevant financial risks while regulating the money supply, solving the financing problems of firms and helping the economy to recover by means of policy guidance and strengthening supervision. On the other hand, the banking industry should strengthen differentiated competition in investment choices, manage risk well and maximize benefits.

The PageRank index in a multi-layer financial network indicates that large state-owned commercial banks have significant systemic importance. Moreover, the more considerable the total amount of trading funds of a bank in a single financial market network, the more trading relationships occur, and when risks occur, it is more likely to lead to cascading defaults by counterparties, and its systemic importance is also higher. Due to their substantial scale, large commercial banks can attract a concentration of trading counterparts and provide a more significant amount of trading capital to the financial markets. Simultaneously, they play the role of credit intermediaries in the interbank market, promoting market equilibrium and thus contributing to financial stability. This study confirms the systemic importance of large commercial banks in the financial system from the perspective of network structure, and the weighted centrality of banks is closely related to their systemic importance. This is another example of “Too central to fail” and that a bank’s transaction behavior and market participation level are the key determinants.

Furthermore, China’s manufacturing, wholesale and retail trade, and real estate industries have higher risk coefficients, among which the cumulative risk of the manufacturing industry is also higher. Meanwhile, the wholesale and retail trade and real estate industries have significantly higher average PageRank indexes, indicating that these industries have higher risk coefficients for a single firm and require more attention to industry risks. Regulatory authorities need to enhance risk monitoring to prevent the aggregation of industry risks. Banks, on their part, must strengthen loan approval and management in critical industries, exercising strict supervision over the use of funds. It was also found that bond investments and trading financial assets have a high-risk coefficient. Therefore, banks and regulators should strictly monitor banks’ investment behavior, guiding banks to engage in diversified investments to prevent large amounts of capital aggregation and systemic risks arising from defaults on single assets.

Based on the stress test results, it was determined that the systemic risks caused by the occurrence of risks in each bank were generally consistent with the systemically important banks identified by the improved PageRank algorithm. The large commercial banks are of significant systemic importance in China’s banking system, which also confirms the applicability of the improved PageRank algorithm proposed in this paper within the multi-layer financial network. Through the comparative analysis of the operational behavior of foreign banks with the six large state-owned commercial banks, it has been found that prudential supervision of the banking system and guiding it towards more rational business activities are crucial measures for managing systemic risks. At the same time, a bank’s systemic importance is closely related to its counterparty risk. Strengthening centralized review before transactions to avoid systemic risk arising from the excessive dependence of a single counterparty on a bank and the over-concentration of risk exposure is also important for managing systemic risk.

This study enriches the related research on systemic risk from the perspective of multi-layer networks. The multi-layer financial network estimation method of China’s banking system and the improved PageRank algorithm presented in this paper serve as references to related research. In addition, this paper identifies systemically important banks, specific industries, and asset classes, and analyzes the factors affecting the systemic importance of banks, which provide a reference for preventing systemic risks.

## Figures and Tables

**Table 1:** Network characteristics of the multi-layer financial network of China’s banking system.

Statistics	Bank–Asset Portfolio Network	Interbank Lending Network	Bank–Firm Credit Network	Multi-Layer Financial Network
Nodes	160	139	1178	1190
Edges	846	424	5923	7193
Density	0.033	0.022	0.004	0.005
Average degree	5.287	3.05	5.028	6.045
Average weighted degree	5.20 × 10^11^	4.56 × 10^10^	1.39 × 10^11^	2.13 × 10^11^
In-degree standard deviations	1.47 × 10^13^	1.98 × 10^11^	5.47 × 10^11^	-
Out-degree standard deviations	1.56 × 10^12^	1.06 × 10^11^	3.19 × 10^12^	-
In-degree power index	1.315	1.297	2.415	-
Out-degree power index	1.613	1.629	1.617	-
In-degree weight power index	1.357	1.625	1.160	-
Out-degree weight power index	1.159	1.483	1.144	-
Average clustering coefficient	0.987	0.656	0.831	0.148

**Table 2:** Top 10 systemically important banks by the PageRank index.

Rank	Multi-Layer Financial Network		Bank–Firm Credit Network		Bank–Asset Portfolio Network		Interbank Lending Network	
	Bank	PageRank	Bank	PageRank	Bank	PageRank	Bank	PageRank
1	ICBC	0.1000	ICBC	0.1009	ICBC	0.0936	ICBC	0.2013
2	ABC	0.0864	CCB	0.0896	ABC	0.0910	ABC	0.1618
3	CCB	0.0860	ABC	0.0818	CCB	0.0782	BCM	0.1016
4	BOC	0.0764	BOC	0.0792	BOC	0.0680	BOC	0.1002
5	BCM	0.0423	BCM	0.0408	PSBC	0.0478	CCB	0.0842
6	CDB	0.0388	CDB	0.0393	BCM	0.0380	CDB	0.0316
7	PSBC	0.0386	PSBC	0.0327	CDB	0.0379	CMB	0.0297
8	CMB	0.0308	CMB	0.0317	CIB	0.0349	PSBC	0.0194
9	CIB	0.0303	CITIC	0.0313	CMB	0.0292	CEXIM	0.0192
10	CITIC	0.0298	SPDB	0.0293	CITIC	0.0280	CMBC	0.0187

ICBC: Industrial and Commercial Bank of China; ABC: Agricultural Bank of China; CCB: China Construction Bank; BOC: Bank of China; BCM: Bank of Communications; CDB: China Development Bank; PSBC: Postal Savings Bank of China; CMB: China Merchants Bank; CIB: Industrial Bank; CITIC: China CITIC Bank; SPDB: Shanghai Pudong Development Bank; CEXIM: The Export-Import Bank of China; CMBC: China Minsheng Bank.

**Table 3:** The top five industries by PageRank index ranking.

Rank	Industries	Total PageRank	Average PageRank	Numbers
1	Manufacturing	0.6383	0.0009	673
2	Wholesale and retail trade	0.0787	0.0018	44
3	Real estate	0.0604	0.0016	38
4	Leasing and business services	0.0532	0.0025	21
5	Information and Technology Services	0.0461	0.0005	100

**Table 4:** The top five asset classes by PageRank index ranking.

Rank	Assets	PageRank	Total Assets
1	Bonds	0.347	5.39 × 10^13^
2	Trading Financial Assets	0.210	1.24 × 10^13^
3	Other Debts	0.130	9.51 × 10^12^
4	Reverse Repurchase Agreements	0.120	5.27 × 10^12^
5	Precious Metals	0.034	8.63 × 10^11^

**Table 5:** Probability of default in the banking system due to different bank failures.

Rank	Banks	Default Probability
1	ICBC	1
2	ABC	1
3	CCB	0.993288591
4	BOC	0.993288591
5	CDB	0.993288591
6	PSBC	0.697986577
7	CIB	0.677852349
8	CMB	0.617449664
9	CITIC	0.590604027
10	SPDB	0.577181208

ICBC: Industrial and Commercial Bank of China; ABC: Agricultural Bank of China; CCB: China Construction Bank; BOC: Bank of China; CDB: China Development Bank; PSBC: Postal Savings Bank of China; CIB: Industrial Bank; CMB: China Merchants Bank; CITIC: China CITIC Bank; SPDB: Shanghai Pudong Development Bank.

**Table 6:** Propagation of failure risk for the six largest state-owned banks.

Banks	Directly Failed Banks	Restricted Banks	Propagation Rounds
ICBC	79	56	4
ABC	58	74	4
CCB	43	77	5
BOC	25	94	5
PSBC	3	92	5
BCM	3	82	3

(ICBC: Industrial and Commercial Bank of China; ABC: Agricultural Bank of China; CCB: China Construction Bank; BOC: Bank of China; PSBC: Postal Savings Bank of China; BCM: Bank of Communications).

## Data Availability

The data used to support the findings of this study are available from the author upon request.
